# The plasma membrane–localized *CsMAC9F1* mediates citrate accumulation in citrus fruit

**DOI:** 10.1093/hr/uhag219

**Published:** 2026-06-03

**Authors:** Yuanyuan Chang, Yuewen Chen, Yushuang Zhao, Feifei Li, Xin Liu, Qiaoying Huang, Ling Sheng, Xiaopeng Lu, Junfeng Yang

**Affiliations:** College of Horticulture, Hunan Agricultural University, 410128 Changsha, China; Fruit Tree Breeding Innovation Center, Yuelushan Laboratory, 410128 Changsha, China; College of Horticulture, Hunan Agricultural University, 410128 Changsha, China; Fruit Tree Breeding Innovation Center, Yuelushan Laboratory, 410128 Changsha, China; College of Horticulture, Hunan Agricultural University, 410128 Changsha, China; Fruit Tree Breeding Innovation Center, Yuelushan Laboratory, 410128 Changsha, China; Fruit Tree Breeding Innovation Center, Yuelushan Laboratory, 410128 Changsha, China; Institute of Horticulture, Hunan Academy of Agricultural Sciences, 410125 Changsha, China; College of Horticulture, Hunan Agricultural University, 410128 Changsha, China; Fruit Tree Breeding Innovation Center, Yuelushan Laboratory, 410128 Changsha, China; College of Horticulture, Hunan Agricultural University, 410128 Changsha, China; Fruit Tree Breeding Innovation Center, Yuelushan Laboratory, 410128 Changsha, China; College of Horticulture, Hunan Agricultural University, 410128 Changsha, China; Fruit Tree Breeding Innovation Center, Yuelushan Laboratory, 410128 Changsha, China; College of Horticulture, Hunan Agricultural University, 410128 Changsha, China; Fruit Tree Breeding Innovation Center, Yuelushan Laboratory, 410128 Changsha, China; College of Horticulture, Hunan Agricultural University, 410128 Changsha, China; Fruit Tree Breeding Innovation Center, Yuelushan Laboratory, 410128 Changsha, China

## Abstract

Citrate, the predominant organic acid in citrus fruits, is a major determinant of fruit acidity. While significant progress has been made in elucidating the mechanisms of citrate accumulation, the comprehensive molecular regulatory network remains incompletely understood. In this study, ‘Jinxiu’ Bingtang sweet orange (*Citrus sinensis*) is a bud mutant derived from ‘Qianyang’ Bingtang sweet orange and exhibits an extremely low-acidity phenotype. *CsMAC9F1* expression was associated with citrate accumulation in both cultivars. Unlike previously characterized tonoplast-localized proteins, CsMAC9F1 localizes to the plasma membrane, suggesting its involvement in a novel pathway regulating citrate accumulation. Functional analyses demonstrated that *CsMAC9F1* acts as a positive regulator of citrate accumulation in citrus. We further identified a bHLH transcription factor, CsbHLH113, which binds the *CsMAC9F1* promoter and activates its expression. Overexpression and silencing analyses across citrus, kumquat, and heterologous tomato systems consistently supported the role of *CsbHLH113* in promoting citrate accumulation through *CsMAC9F1*. Together, our findings reveal a CsbHLH113–*CsMAC9F1* regulatory module that adds a plasma membrane–associated branch to the citrus acidity network and provides new insights into the transcriptional control of fruit acidity.

## Introduction

Citrus fruit acidity is a major determinant of eating quality. Citrate is the predominant organic acid in citrus juice sacs, accounting for more than 90% of total organic acids [1, 2]. Significant variation in citrate content, both between and within species, underlies the common classification of citrus cultivars into high- and low-acid types [3, 4], highlighting the genetic complexity in the regulation of citrate metabolism.

In plant cells, mitochondria do not accumulate high levels of citrate, and the cytosol is maintained at a near-neutral pH; thus, the acidic vacuole serves as the primary organelle for citrate sequestration. Consequently, vacuolar transport represents a critical control point for fruit acidity [5, 6]. As a hydrophilic molecule, citrate cannot passively diffuse across the tonoplast; its transport is an active process dependent on the proton motive force generated by tonoplast-localized proton pumps. V-type H^+^-ATPases (V-ATPase), H^+^-pyrophosphatase (V-PPase), and P-type H^+^-ATPases establish and maintain the transmembrane proton gradient, thereby providing the driving force for citrate sequestration into the vacuole [7–9].

Tonoplast-localized P_3A_-type ATPases have been extensively implicated in vacuolar acidification and citrate accumulation [2, 4, 10]. In petunia, *PhPH5* is a core regulator of vacuolar acidification in petals [11], and a homologous P_3A_-ATPase in tomato acidifies petal vacuoles and influences flower coloration [12]. In citrus, the *PhPH5* homolog *CsPH8* is preferentially expressed in high-acid varieties and has been functionally validated as a key determinant of citrate accumulation [4, 13].

In citrus, multiple acidity regulators converge on the transcriptional control of vacuolar acidification. CsPH8, encoding a vacuolar P-type proton pump, is regulated by a WRKY–WD40–R2R3-MYB–bHLH (WWMB) complex that integrates developmental and environmental cues to modulate citrate storage [14, 15]. In addition, the R2R3-MYB factor PH4 interacts with the bHLH protein AN1 and a WD40 co-activator to coordinately regulate anthocyanin biosynthesis and vacuolar acidification [16–18]. Notably, the AN1-like bHLH transcription factor Noemi is a major genetic determinant of the acidless phenotype, underscoring the central role of bHLH-centered transcriptional regulation in citrus acidity formation [4]. Despite these advances, current models of citrus acidity regulation are largely centered on nuclear transcriptional complexes and tonoplast-localized proton pumps. How bHLH transcription factors connect transcriptional regulation to downstream effector components remains incompletely understood.

In addition to these established regulatory mechanisms, *MAC9F1* has also been proposed as a gene associated with vacuolar acidification. In petunia, *PhMAC9F1* was first identified in expression screens for AN1/PH3/PH4-dependent transcripts and was found to be downregulated in *an1* mutant petals. Moreover, RNA interference (RNAi)–mediated silencing of *PhMAC9F1* resulted in a blue-flower phenotype accompanied by increased vacuolar pH in petal epidermal cells, supporting a role for *PhMAC9F1* in vacuolar lumen acidification. In citrus, the homolog *CitMAC9F1* shows a strong association with fruit acidity. In the lemon cultivar ‘Faris’, *CitMAC9F1* is highly expressed in juice vesicles of sour fruits (Fso) but is markedly reduced in sweet fruits (Fsw), and its expression parallels that of *CitPH1* and *CitPH5* and is tightly associated with fruit pH and citrate content [4]. More recently, in sweet orange, *CsMAC9F1* was identified as part of the drought-responsive CsABF3–CsAN1/*CsPH8* module. It is transcriptionally activated by *CsAN1*, promotes citrate accumulation upon transient overexpression, and may partially compensate for reduced *CsPH8* function [13]. However, how *CsMAC9F1* is transcriptionally regulated and how it contributes to citrate accumulation remain unclear.

In this work, we investigated the role of *CsMAC9F1* in regulating citrus fruit acidity. We hypothesized that *CsMAC9F1* acts downstream in the regulation of citrate accumulation and is transcriptionally activated by a basic helix–loop–helix (bHLH) factor. Using ‘Qianyang’ Bingtang sweet orange (*Citrus sinensis*) and its extremely low-acid mutant ‘Jinxiu’, we found that *CsMAC9F1* expression is associated with citrate accumulation. Overexpression assays in multiple plant materials further demonstrated that *CsMAC9F1* positively regulates citrate levels. By yeast one-hybrid (Y1H) screening, we identified *CsbHLH113* as a transcriptional activator that binds the *CsMAC9F1* promoter and activates its expression. Together, these results define a CsbHLH113–*CsMAC9F1* regulatory module that promotes citrate accumulation in citrus fruit and expands the transcriptional network underlying citrus acidity.

## Results

### Characterization of acid levels in ‘Jinxiu’ Bingtang sweet orange

‘Jinxiu’ Bingtang sweet orange (*C. sinensis*; hereafter ABT) is an elite bud mutant of ‘Qianyang’ Bingtang sweet orange (*C. sinensis*; hereafter BT) (Fig. 1A). The most prominent phenotypic difference between the two cultivars is fruit acidity, with ABT consistently exhibiting lower organic acid content (Fig. 1B). Citrate levels in ABT fruit declined markedly from 90 to 150 days after full bloom (DAFB), whereas those in BT fruit remained consistently high. Consequently, citrate concentrations were 33%–69% lower in ABT than in BT during this developmental period (Fig. 1C). This reduction was accompanied by an increase of 0.5–0.8 pH units in ABT relative to BT (Fig. 1D). Total soluble solids (TSS) in ABT fruit were significantly higher than in BT, representing a 26% increase (Fig. 1E).

**Figure 1:**
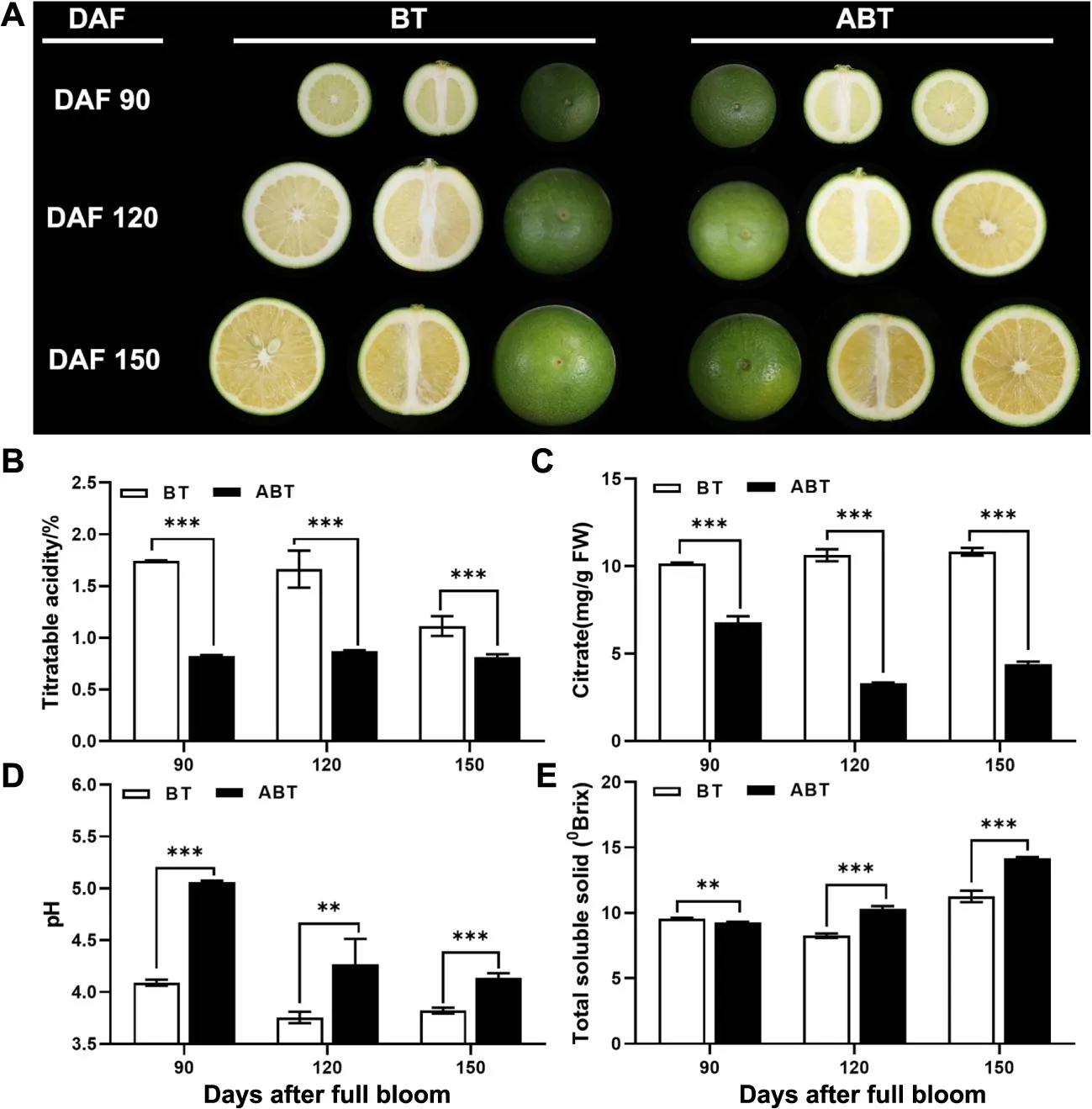
The ABT bud mutant exhibits characteristically low acidity coupled with elevated total soluble solids (TSSs). (A) Fruits of BT and ABT harvested at 90, 120, and 150 days after full bloom (DAFB). Scale bar = 2 cm. (B) Titratable acid (TA) content in fruits at 90, 120, and 150 DAFB. (C) Dynamic changes in citrate concentration during fruit development from 90, 120, and 150 DAFB. (D) Fruit pH values at 90, 120, and 150 DAFB. (E) TSS content in fruits at 90, 120, and 150 DAFB. Error bars represent SD (*n* = 3). ^*^P < 0.05; ^**^P < 0.01; ^***^P < 0.001.

### *CsMAC9F1* expression is tightly associated with citrate accumulation in citrus fruit

To elucidate the molecular basis of citrate metabolism in citrus, we performed RNA-seq of BT and ABT fruits at three developmental stages (July, August, and September; hereafter stages 7, 8, and 9) (Table S1). Principal component analysis (PCA) showed a clear separation between BT and ABT samples (Fig. 2A). To identify candidate genes associated with fruit acidity, we integrated multiple transcriptomic datasets, including pairwise comparisons between BT and ABT at each stage (BT7_vs_ABT7, BT8_vs_ABT8, BT9_vs_ABT9), transcriptomes from drought-stressed ‘Bingtang’ sweet orange and corresponding controls at the September fruit expansion stage (DT9_vs_CK9; DT9, high acid; CK9, low acid) (Table S2) [13], and a public dataset from high- and low-acid pummelo (HCA_vs_LCA) [18]. Intersection of these five comparisons identified 23 core genes that were consistently differentially expressed (Fig. 2B).

**Figure 2:**
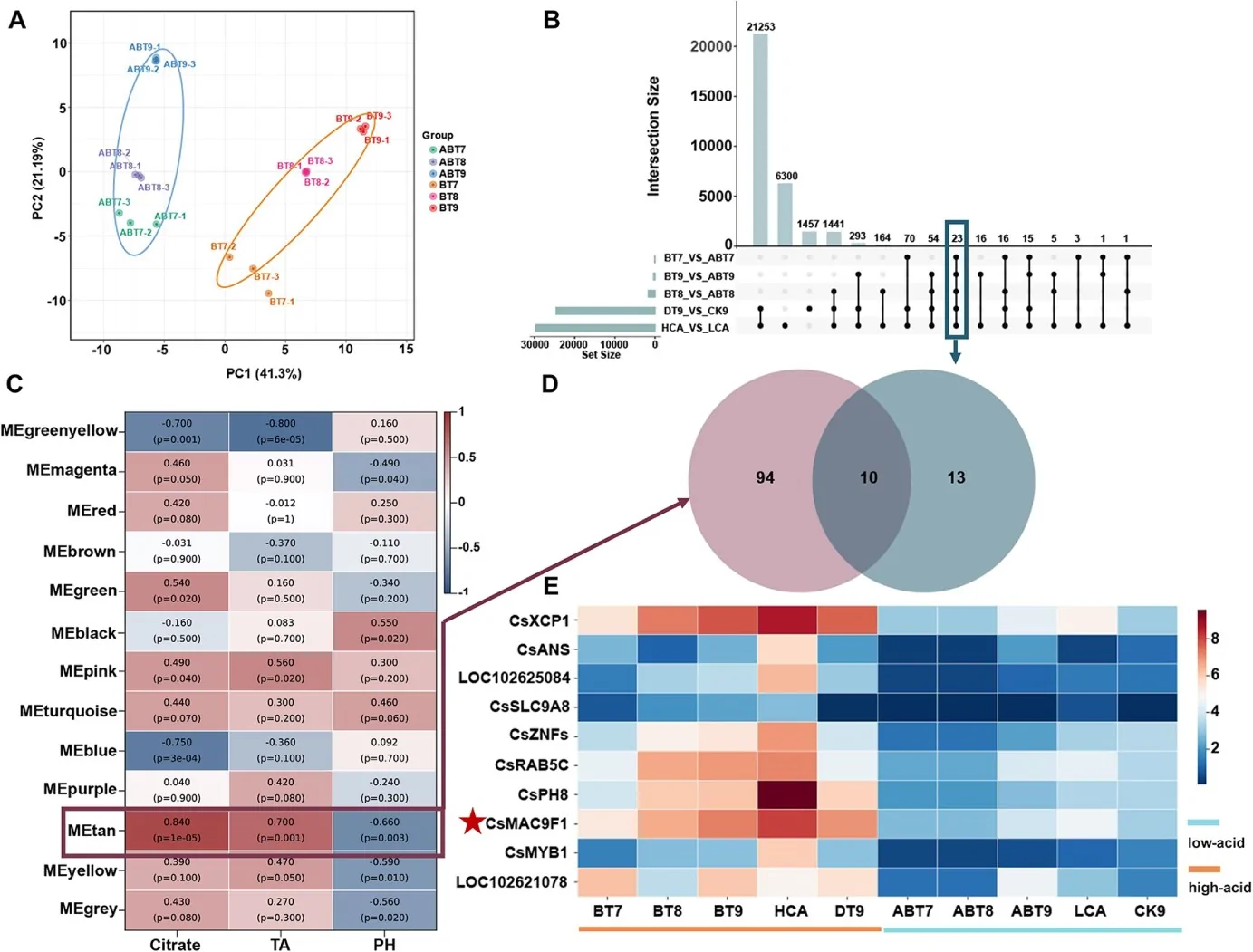
Identification of *CsMAC9F1* as a candidate gene associated with fruit acidity by integrative transcriptomic analyses. (A) Principal component analysis (PCA) of transcriptomic profiles from BT and the low-acid mutant (ABT) fruit, illustrating a clear separation between BT and ABT samples consistent with the acid phenotype. (B) Venn diagram identifying a core set of 23 shared differentially expressed genes associated with fruit acidity through intersection analysis. The five transcriptomic comparisons span fruit developmental stages (BT vs. ABT), drought stress (DT9 vs. CK9), and diverse genetic backgrounds (pummelo; HC vs. LCA). (C) Weighted gene co-expression network analysis (WGCNA) of the developmental time-series transcriptome identified the MEtan module as the module most significantly associated with fruit acidity. (D) Integration of WGCNA and intersection analysis. Genes from the MEtan module (104 genes) were intersected with the 23 core differentially expressed genes from (B), identifying 10 overlapping candidate genes. (E) Expression profiling of the 10 core candidate genes across multiple datasets. The expression pattern of the novel candidate *CsMAC9F1* is highly concordant with that of *CsPH8*, a key regulator of citrate accumulation.

To further pinpoint key regulators of citrate metabolism, weighted gene co-expression network analysis (WGCNA) was performed, revealing 13 distinct co-expression modules (Fig. 2C). Among these, the MEtan module exhibited the strongest correlation with acidity content and comprised 104 genes (Fig. 2C; Table S3). Intersecting the genes in this module with the 23 core differentially expressed genes yielded 10 overlapping candidates (Fig. 2D). Across multiple datasets, *CsMAC9F1* showed high expression, strong co-expression with the key citrate regulator *CsPH8*, and a close association with citrate content (Fig. 2E; Fig. S1; Table S4), suggesting that *CsMAC9F1* expression is closely associated with citrate accumulation in citrus fruit.

CsMAC9F1 encodes a protein homologous to petunia PhMAC9F1. CsMAC9F1 is annotated as gene model Cs1g26060 in the sweet orange reference genome and encodes the protein KAH9710771.1. Unlike the well-characterized tonoplast-localized vacuolar acidification regulators PH1 and VHP, *CsMAC9F1* may represent a distinct factor involved in citrate accumulation in citrus. Multiple sequence alignment and an AlphaFold-predicted structural model revealed a highly conserved motif shared by MAC9F1 proteins (Fig. 3A). Phylogenetic analysis grouped CsMAC9F1 with its orthologs from apple (MdMAC9F1) and petunia (PhMAC9F1), indicating close evolutionary relatedness (Fig. 3B). The widespread presence of MAC9F1 orthologs across diverse plant species suggests a conserved role in pH and acid homeostasis.

**Figure 3:**
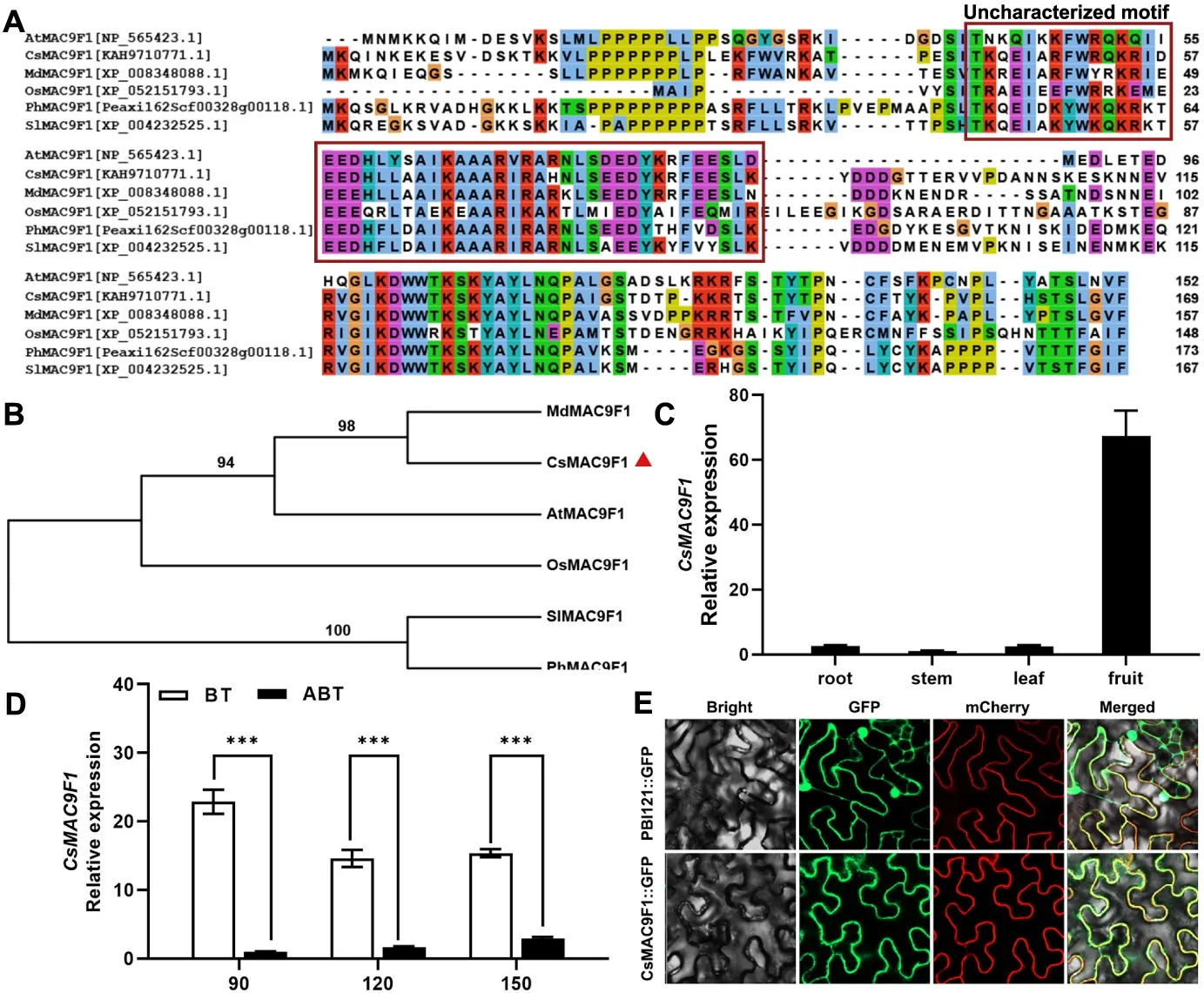
Sequence features, phylogeny, expression pattern, and subcellular localization of CsMAC9F1. (A) Multiple sequence alignment of CsMAC9F1 and MAC9F1 homologs from representative plant species. The conserved uncharacterized motif is indicated by a box; motif boundaries were inferred from the AlphaFold Protein Structure Database (https://alphafold.ebi.ac.uk/). Protein accessions used in the alignment are: CsMAC9F1 (*C. sinensis*, KAH9710771.1; gene model Cs1g26060 in C. sinensis v1.0), AtMAC9F1 (*A. thaliana*, NP_565423.1), MdMAC9F1 (*M. domestica*, XP_008348088.1), OgMAC9F1 (*O. glaberrima*, XP_052151793.1), SlMAC9F1 (*S. lycopersicum*, XP_004232525.1), and PhMAC9F1 (*Petunia axillaris*, Peaxi162Scf00328g00118.1). (B) Neighbor-joining phylogenetic tree of MAC9F1 proteins. The *O. glaberrima* RefSeq protein XP_052151793.1 was used to represent the *Oryza* homolog because the corresponding *O. sativa* sequence in NCBI is partial. (C) Tissue-specific expression of CsMAC9F1 in citrus. (D) CsMAC9F1 expression during fruit development (90–150 DAFB) in BT and ABT. (E) Subcellular localization of CsMAC9F1–GFP in *N. benthamiana* epidermal cells. PM, plasma membrane marker (mCherry). Error bars represent SD (*n* = 3). ^*^P < 0.05; ^**^P < 0.01; ^***^P < 0.001.

*CsMAC9F1* was predominantly expressed in fruit tissues, with much lower transcript levels in roots and leaves (Fig. 3C). During fruit development (90–150 DAFB), *CsMAC9F1* expression increased substantially in BT fruits but remained low in ABT fruits (Fig. 3D). Subcellular localization analysis in tobacco epidermal cells showed that CsMAC9F1 predominantly colocalized with a plasma membrane marker, supporting its plasma membrane localization (Fig. 3E).

### *CsMAC9F1* promotes citrate accumulation in citrus

To assess the function of *CsMAC9F1* in citrate metabolism, we generated stable *CsMAC9F1*-overexpressing citrus calli (Fig. 4A). Two independent lines (MAC9F1-OE24 and MAC9F1-OE28) showed markedly elevated CsMAC9F1 transcript levels relative to the wild type (WT) control (Fig. 4B). In parallel, citrate accumulation was increased in both overexpression callus lines (Fig. 4C). We also generated *CsMAC9F1*-overexpressing citrus plants (OE-1 and OE-2) (Fig. 4D). In these lines, *CsMAC9F1* transcript levels were significantly higher than those in the empty-vector (EV) controls, and citrate content was correspondingly increased (Fig. 4E and F). Similar results were obtained in transient overexpression assays in citrus juice sacs, in which CsMAC9F1 upregulation was associated with increased citrate accumulation (Fig. S2).

**Figure 4:**
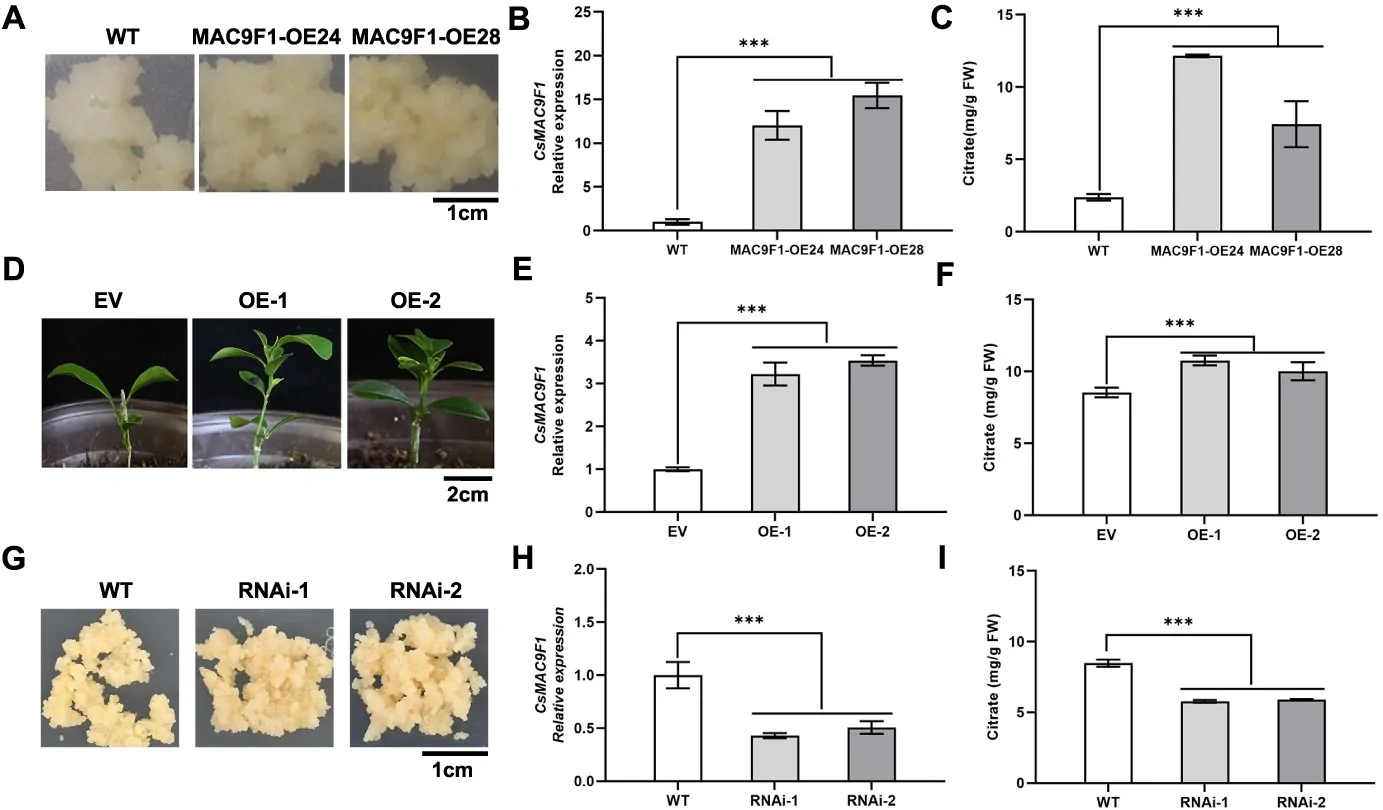
Citrate accumulation in *CsMAC9F1*-transgenic citrus calli and seedlings. (A) Phenotype of wild-type (WT) and two independent *CsMAC9F1*-overexpressing (OE) transgenic calli lines. (B and C) Analysis of *CsMAC9F1* expression and citrate accumulation in overexpressing calli lines. Wild-type (WT) calli served as control. (D) Phenotype of empty-vector (EV) control and two stable *CsMAC9F1*-OE transgenic citrus seedlings. (E and F) Relative expression of *CsMAC9F1* and citrate content in *CsMAC9F1*-overexpressing and control seedlings. (G) Phenotype of WT and two *CsMAC9F1*-RNAi transgenic calli lines. (H and I) RNA interference (RNAi) was performed to suppress *CsMAC9F1* expression, resulting in reduced citrate accumulation in calli. Error bars represent SD (*n* = 3). ^*^P < 0.05; ^**^P < 0.01; ^***^P < 0.001.

To further test the requirement of CsMAC9F1, we next used RNAi to suppress endogenous *CsMAC9F1*. Two independent silenced lines (RNAi-1 and RNAi-2) showed strongly reduced *CsMAC9F1* expression (Fig. 4G). Consistent with the gain-of-function results, both RNAi lines exhibited significantly decreased citrate contents (Fig. 4H and I). In addition, transient silencing assays in citrus calli showed a similar trend, with *CsMAC9F1* silencing resulting in a significant reduction in citrate content (Fig. S3). These results indicate that *CsMAC9F1* positively contributes to citrate accumulation in citrus.

Together, gain- and loss-of-function analyses in citrus calli, juice sacs, and whole plants demonstrate that *CsMAC9F1* acts as a positive regulator of citrate accumulation.

### CsbHLH113 activates *CsMAC9F1* by binding to its promoter

To identify upstream regulators of CsMAC9F1, we performed a Y1H screen using the 2-kb promoter region of CsMAC9F1 as bait. For Y1H analysis, the promoter was divided into a distal fragment (−2000 bp to −1000 bp) and a proximal fragment (−1000 bp to −1 bp). CsbHLH113 was identified as a candidate regulator, and targeted Y1H assays further showed that yeast cells co-expressing CsbHLH113 and the proximal promoter fragment were able to grow on selective medium containing 200 ng/ml Aureobasidin A (AbA), whereas no growth was observed with the distal fragment (Fig. 5A and B). These results indicate that CsbHLH113 interacts with the proximal region of the CsMAC9F1 promoter in yeast.

**Figure 5:**
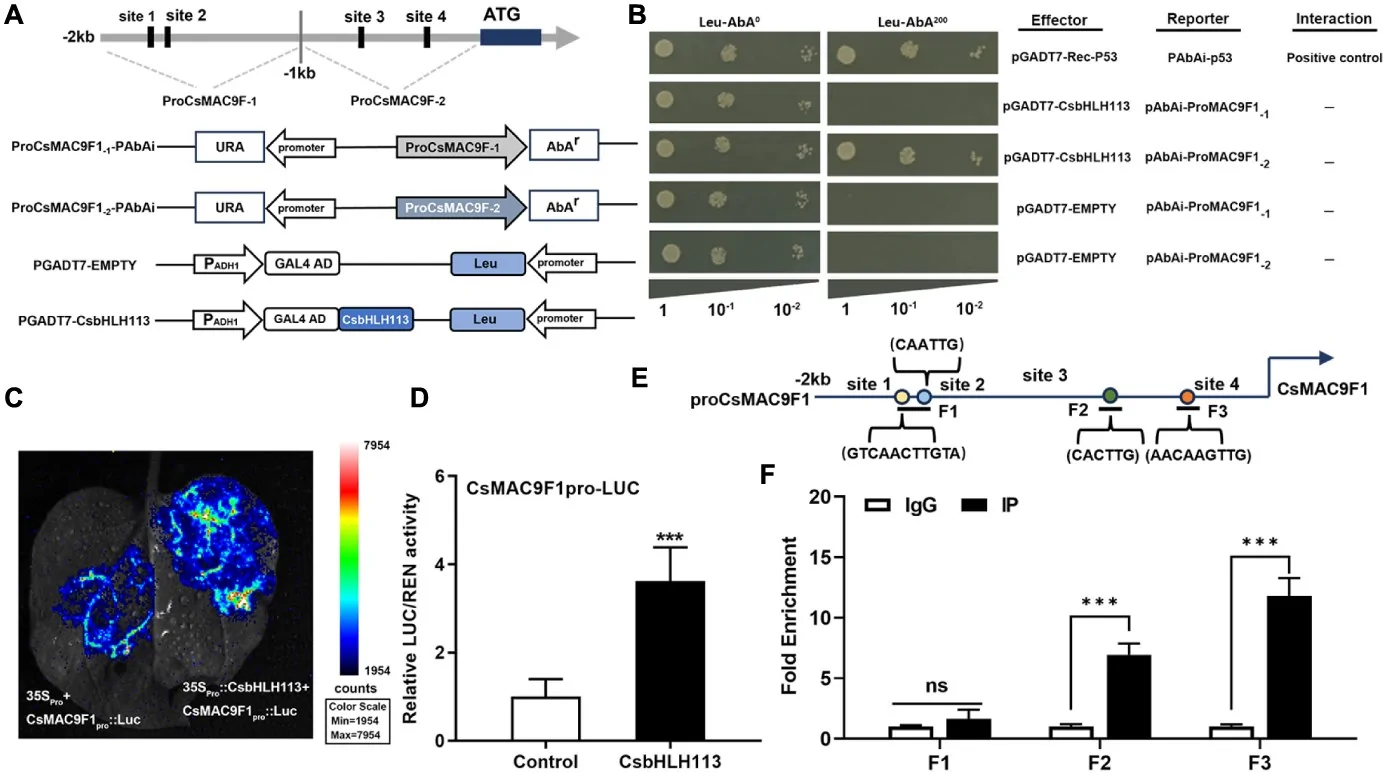
Molecular characterization of CsbHLH113 binding to and activating the *CsMAC9F1* promoter. (A) Schematic diagram of the CsMAC9F1 promoter showing the predicted CsbHLH113-binding sites. (B) Targeted Y1H assay showing that yeast cells co-expressing CsbHLH113 with the proximal *CsMAC9F1* promoter fragment (−1000 to −1 bp) grew on selective medium containing 200 ng/ml AbA, whereas no growth was observed with the distal fragment (−2000 to −1000 bp). Positive and negative controls were included. (C) Luminescence image from a dual-luciferase assay in *N. benthamiana* leaves. (D) Quantification of LUC/REN ratios demonstrating significant promoter activation by CsbHLH113 compared to the empty vector (EV) control. (E) Schematic representation of the *CsMAC9F1* promoter showing the predicted *CsbHLH113* binding motifs and the regions (F1, F2, and F3) analyzed by ChIP–qPCR. (F) ChIP–qPCR analysis showing *CsbHLH113* enrichment at the *CsMAC9F1* promoter in citrus calli expressing 35S::*CsbHLH113*–EGFP. Chromatin was immunoprecipitated using an anti-GFP antibody (IP), while a nonspecific IgG antibody served as the negative control. The relative enrichment of the F1, F2, and F3 fragments was quantified by qPCR. Error bars represent SD of three biological replicates. Asterisks indicate significant differences compared with the IgG control (^*^P < 0.05; ^**^P < 0.01; ^***^P < 0.001).

To determine whether CsbHLH113 regulates *CsMAC9F1* transcription in planta, we performed a transient dual-luciferase assay in *N. benthamiana* leaves. An effector construct (35Spro::CsbHLH113) was co-infiltrated with a reporter construct in which the 2-kb CsMAC9F1 promoter drove firefly luciferase (LUC). Co-expression of CsbHLH113 significantly increased the LUC/REN ratio by ~3.6-fold relative to the empty-vector control (Fig. 5C and D), indicating that CsbHLH113 activates the CsMAC9F1 promoter.

To further examine whether CsbHLH113 is associated with the CsMAC9F1 promoter *in vivo*, we performed chromatin immunoprecipitation coupled with quantitative polymerase chain reaction (ChIP-qPCR) using citrus calli expressing 35S::*CsbHLH113*–GFP. Two promoter regions (F1 and F2) containing putative E-box motifs were selected for enrichment analysis (Fig. 5E). ChIP-qPCR showed that both F1 and F2 were significantly enriched in the anti-GFP immunoprecipitated samples relative to the IgG control (Fig. 5F), supporting the *in vivo* association of CsbHLH113 with the CsMAC9F1 promoter.

Together, these results support the conclusion that CsbHLH113 is associated with the CsMAC9F1 promoter and positively regulates its transcription.

### CsbHLH113 regulates citrate accumulation via *CsMAC9F1*

To characterize *CsbHLH113*, we first performed bioinformatic analyses, which identified a canonical bHLH domain with conserved residues required for DNA binding and dimerization (Fig. 6A). Phylogenetic analysis showed that *CsbHLH113* clustered with *MibHLH153* from *Mangifera indica*, suggesting potential functional conservation (Fig. 6B).

**Figure 6:**
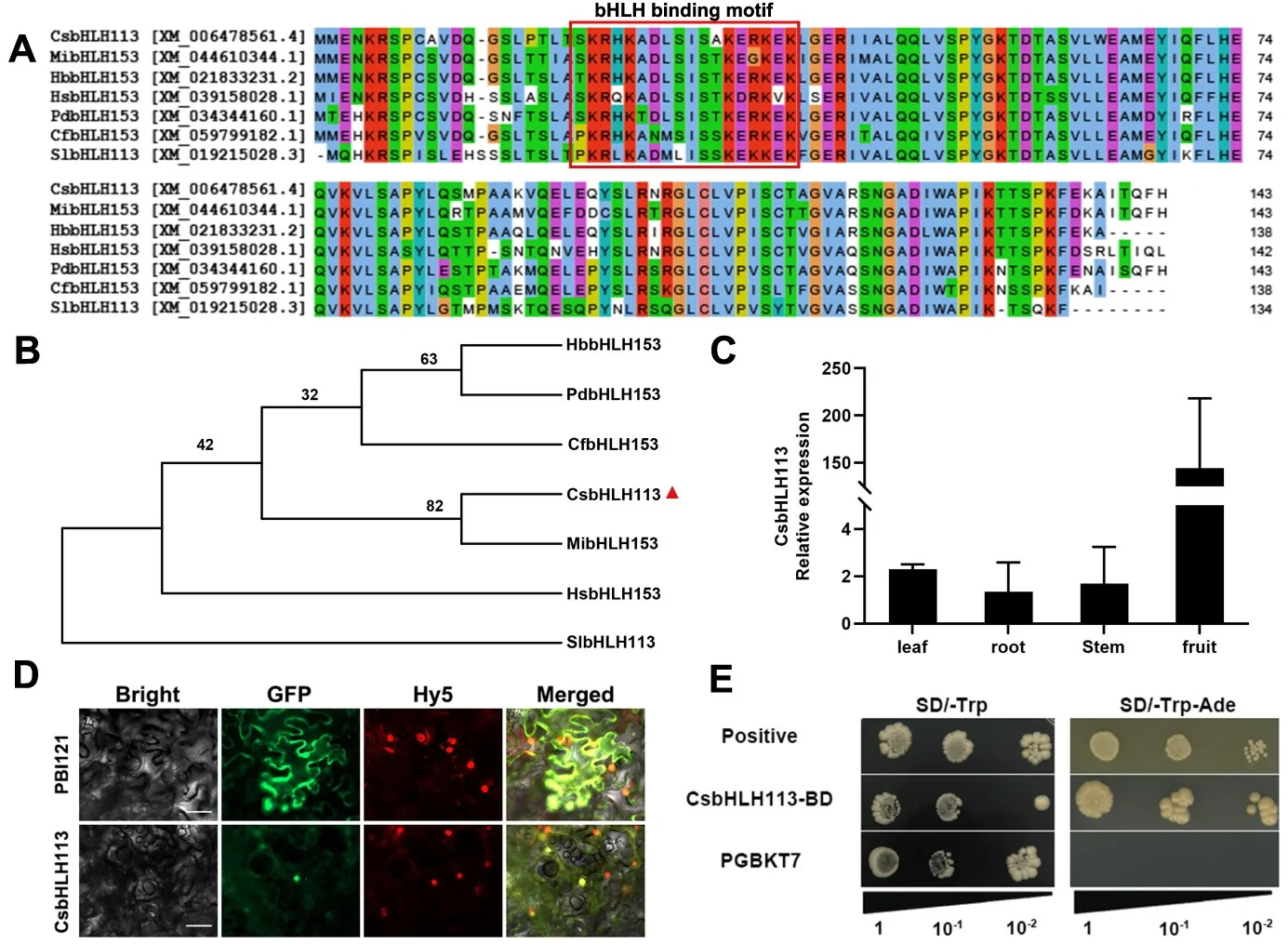
Functional characterization of CsbHLH113 as a transcriptional activator in citrus. (A) The conserved bHLH domain in CsbHLH113 and related proteins. Conserved residues are boxed. (B) Phylogenetic analysis showing the evolutionary relationship of CsbHLH113 with other bHLH proteins. (C) Tissue-specific expression patterns of CsbHLH113 in different citrus organs. (D) Subcellular localization of the CsbHLH113-GFP fusion protein in *N. benthamiana* epidermal cells. Scale bar = 20 μm. (E) Transcriptional activation assay in yeast. Growth of yeast cells carrying pGBKT7-CsbHLH113 on SD/-Trp-Ade selective medium, demonstrating the transcriptional activation capability of CsbHLH113. Error bars represent SD (*n* = 3).

Expression profiling analysis showed that *CsbHLH113* was predominantly expressed in fruit, with moderate levels in leaves and minimal expression in other tissues (Fig. 6C). Transient expression of CsbHLH113–EGFP in *N. benthamiana* indicated exclusive nuclear localization (Fig. 6D). In yeast, CsbHLH113 exhibited transactivation activity, as cells harboring pGBKT7-CsbHLH113 grew on selective medium whereas the empty-vector control did not (Fig. 6E). These results establish that CsbHLH113 is a nuclear-localized transcriptional activator with preferential expression in fruit.

To determine the role of *CsbHLH113* in citrate accumulation, we performed gain- and loss-of-function analyses. Two independent citrus callus lines overexpressing *CsbHLH113* (OE-7 and OE-9) were generated (Fig. 7A). Both lines showed ~6-fold increase in *CsbHLH113* transcript abundance (Fig. 7B) and accumulated significantly more citrate (2.5-fold) than the control (Fig. 7C). The transcript level of the downstream gene *CsMAC9F1* was also elevated in the overexpression lines (Fig. S4), supporting a positive association between *CsbHLH113* expression and *CsMAC9F1* transcription.

**Figure 7:**
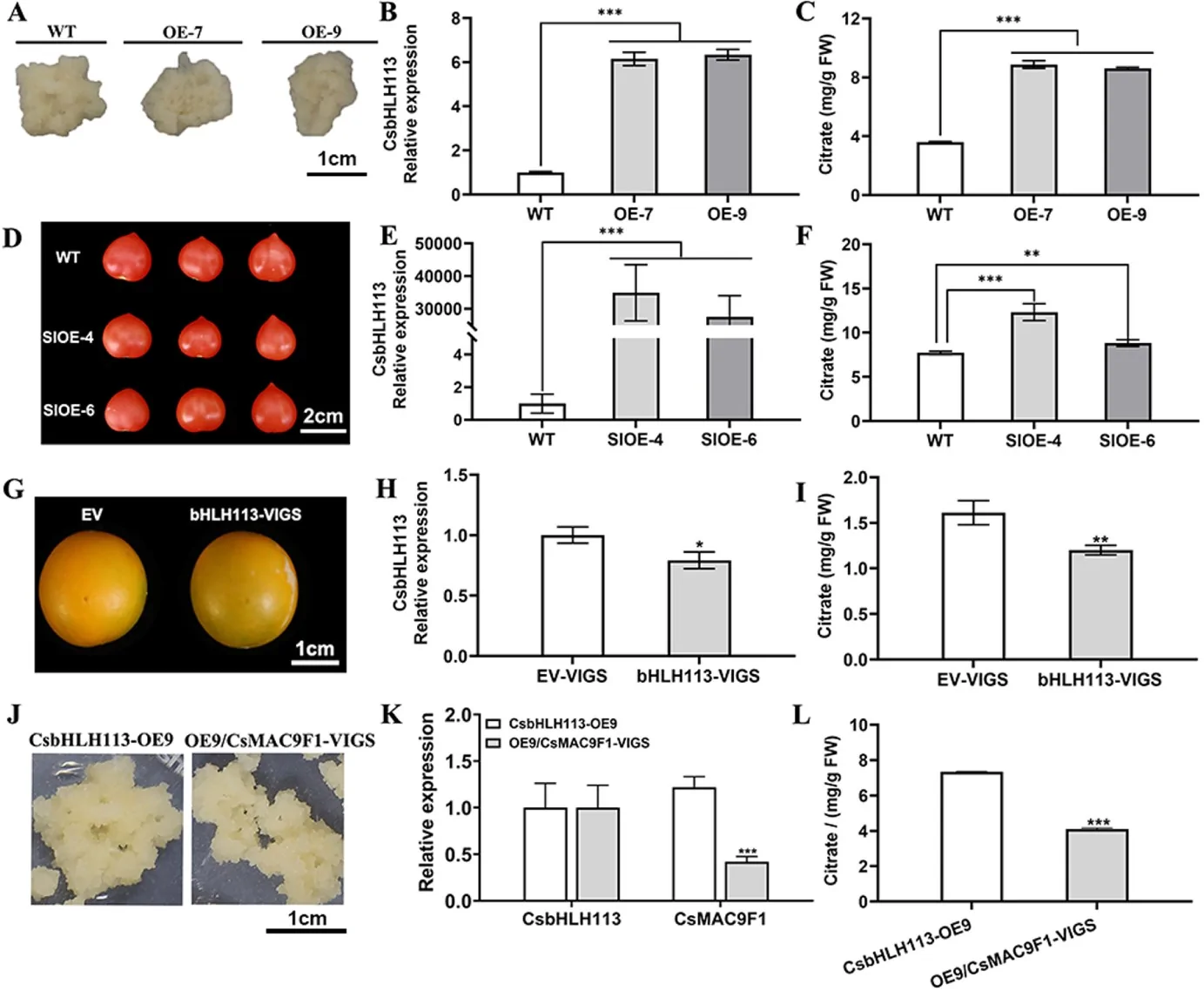
*CsbHLH113* positively regulates citrate accumulation. (A) Representative image of wild-type (WT) and CsbHLH113-overexpressing (OE) citrus calli. (B) Expression levels of *CsbHLH113* in *CsbHLH113*-OE citrus calli. (C) Citrate content in CsbHLH113-OE citrus calli. (D) CsbHLH113-OE tomatoes. (E) Expression levels of *CsbHLH113* in CsbHLH113-OE tomatoes. (F) Citrate content in CsbHLH113-OE tomato. (G) Kumquat fruits with *CsbHLH113* silencing. (H) Expression levels of CsbHLH113 in kumquat fruits subjected to virus-induced gene silencing (VIGS). (I) Citrate content in kumquat fruits with VIGS-mediated CsbHLH113 silencing. (J) Transient silencing of *CsMAC9F1* in CsbHLH113-overexpressing citrus callus (OE-9). (K) Relative expression levels of *CsMAC9F1* were determined following infection with either a *CsMAC9F1*-VIGS construct or an empty vector (EV) control. (L) Citrate content in OE-9 calli infected with the EV control or the *CsMAC9F1*-VIGS construct. Error bars represent SD (*n* = 3). ^*^P < 0.05; ^**^P < 0.01; ^***^P < 0.001.

To assess functional conservation, we constitutively overexpressed *CsbHLH113* in tomato (Fig. 7D). The transgene was expressed at very high levels (27 000–35 000-fold higher than the endogenous tomato transcript level) (Fig. 7E). This overexpression led to a consistent increase in fruit citrate content (1.2- to 1.6-fold) and was associated with earlier flowering and fruit set (Fig. 7F; Fig. S5), suggesting that *CsbHLH113*-mediated regulation of citrate accumulation may be conserved across species.

To test whether *CsbHLH113* is required for normal citrate accumulation, we suppressed its expression in kumquat fruit using virus-induced gene silencing (VIGS) (Fig. 7G). *CsbHLH113* transcript levels were reduced by 70%–85% in independent silencing samples (Fig. 7H). This reduction was accompanied by decreased *CsMAC9F1* expression and a significant 25%–30% decrease in citrate concentration (Fig. 7I; Figs S2 and S3). Together, these complementary results indicate that *CsbHLH113* functions as a positive regulator of citrate accumulation.

To delineate regulatory relationships within the pathway, we analyzed the expression of *CsPH8* and *CsbHLH113* in *CsMAC9F1*-overexpressing citrus seedlings and in calli subjected to transient *CsMAC9F1* silencing. *CsPH8* transcript levels were upregulated in overexpression lines and downregulated upon *CsMAC9F1* silencing, whereas *CsbHLH113* expression was largely unchanged (Fig. S7), consistent with *CsMAC9F1* acting downstream of *CsbHLH113*.

Finally, we performed epistasis analysis by silencing *CsMAC9F1* in the high-citrate *CsbHLH113*-overexpressing callus line OE-9. OE-9 calli infiltrated with the VIGS empty vector (TRV control) retained the high-citrate phenotype, whereas VIGS-mediated *CsMAC9F1* silencing markedly reduced citrate accumulation (Fig. 7J–L). These data demonstrate that *CsMAC9F1* is required for *CsbHLH113*-driven citrate accumulation, genetically placing it downstream in the pathway.

## Discussion

Citrate is the predominant organic acid in citrus fruit and a key determinant of acidity, flavor, and consumer preference [19]. Following its synthesis in the mitochondrial tricarboxylic acid cycle, citrate is transported into the vacuole, where it accumulates to high levels. This process is coupled with vacuolar acidification, primarily driven by proton pumps such as V-ATPase, V-PPase, and P-type H^+^-ATPases [5, 20, 21]. The activity of these key acidification-related enzymes is tightly regulated by core transcriptional machinery operating in the nucleus and at the tonoplast. For instance, in citrus, the transcription factors *PH3* (WRKY) and *PH4* (MYB) form a nuclear complex with the bHLH protein AN1 and the WD-repeat protein AN11, which, in turn, activates the expression of the tonoplast P3A-type H^+^-ATPase gene *PH8* (a homolog of petunia *PH5*), thereby promoting vacuolar acidification [11]. Similarly, in petunia, the loss of PH5 function compromises the active proton transport capacity across the tonoplast, reducing the efficiency of proton influx into the vacuolar lumen. As a result, the proton gradient across the vacuolar membrane fails to be maintained, leading to reduced vacuolar acidity. The decreased acidity alters the molecular structure of anthocyanins, shifting petal coloration from red/violet to blue and further impairing anthocyanin stability and accumulation [22]. Although previous studies have largely focused on nuclear transcriptional complexes and tonoplast proton pumps, citrate accumulation is likely influenced by multiple membrane-associated processes, including not only tonoplast acidification but also cytosolic pH homeostasis and ion/proton balance across other cellular membranes. Therefore, plasma membrane-localized components may also contribute to fruit acidity by indirectly affecting the cellular conditions required for vacuolar citrate sequestration.

Although the molecular function of MAC9F1 proteins remains unclear, accumulating evidence from multiple species supports a conserved association between MAC9F1 homologs and cellular-acid/organic-acid homeostasis. In petunia, *PhMAC9F1* was identified as an *AN1/PH3/PH4*-dependent transcript, and RNAi-mediated suppression of *PhMAC9F1* increases vacuolar pH and results in a blue-flower phenotype, consistent with impaired vacuolar acidification [22]. In citrus, *CitMAC9F1/CsMAC9F1* expression correlates with fruit acidity and citrate accumulation [4, 13]. Our transcriptomic analyses further revealed a strong association between *CsMAC9F1* expression and fruit acidity (Fig. 2; Fig. S1). Functional assays demonstrated that *CsMAC9F1* positively regulates citrate accumulation across multiple experimental systems, and its expression changes coordinately with the vacuolar acidification–related gene *CsPH8* (Fig. 4; Figs S2, S3, and  S7). These observations suggest that, although CsMAC9F1 is not a tonoplast protein, it may still be functionally connected to cellular processes that support vacuolar citrate storage and acidification. Because vacuolar citrate accumulation depends not only on tonoplast proton pumps but also on the broader intracellular proton and ion balance, changes at the plasma membrane could indirectly influence the conditions required for efficient citrate sequestration into the vacuole. An additional observation in our study is that perturbation of *CsMAC9F1* expression was accompanied by corresponding changes in the transcript abundance of *CsPH8* (Fig. S7), a core component of the tonoplast acidification machinery. Given that CsMAC9F1 localizes to the plasma membrane (Fig. 3E), it is unlikely to act as a direct regulator of *CsPH8*. Instead, we propose that *CsMAC9F1* may influence *CsPH8* expression indirectly, potentially through feedback regulation associated with cellular ion balance, proton motive force, or cytosolic pH homeostasis. Thus, our current data support a model in which *CsMAC9F1* functions in a plasma membrane–associated regulatory layer that is parallel to, yet functionally coupled with, the canonical *CsPH8*-centered vacuolar acidification pathway, rather than acting as a direct upstream regulator of *CsPH8*. These results indicate that *CsMAC9F1* acts as a positive regulator of citrate accumulation and acidity in sweet orange fruit.

A key feature distinguishing *CsMAC9F1* from most known acidity regulators is its plasma membrane localization. Increasing evidence indicates that plasma membrane transporters and proton pumps can substantially influence intracellular organic-acid homeostasis by shaping proton motive force (PMF), membrane potential, and cytosolic pH. For example, in maize, the plasma membrane transporter *ZmMATE1* exploits external proton gradients to drive H^+^ influx coupled to citrate^−^ antiport, resulting in net citrate efflux into the rhizosphere and altered citrate distribution at the tissue level [23]. The plasma membrane H^+^-ATPase is the major outward proton pump in plant cells and establishes the PMF that underpins secondary transport; sustained proton extrusion stabilizes cytosolic pH and acidifies the apoplast, thereby supporting diverse physiological processes [5, 24, 25]. Likewise, *AtALMT1* is activated by Al^3+^ and mediates malate efflux along existing electrochemical gradients; this response can stimulate H^+^-ATPase activity to maintain membrane potential and induce metabolic compensation through upregulation of PEPC and MDH to replenish organic-acid pools [26]. These examples illustrate that plasma membrane–localized factors can influence organic-acid distribution and accumulation by modulating proton gradients, membrane potential, and cytosolic pH, even when the final storage site of organic acids is the vacuole. In our study, confocal imaging showed that CsMAC9F1 co-localizes with a plasma membrane marker (Fig. 3E), and genetic perturbations of *CsMAC9F1* consistently altered citrate accumulation (Fig. 4; Figs S2 and S3). Together, these findings support the contribution of plasma membrane–localized factors to the control of fruit organic-acid accumulation, including citrate.

These findings raise an important question regarding how this plasma membrane–associated regulatory layer is transcriptionally coordinated during citrate accumulation. bHLH transcription factors constitute a central regulatory layer in organic-acid metabolism across multiple fruit crops. In apple, *MdbHLH3* and *MdbHLH1* positively regulate malate and citrate accumulation by activating metabolic genes and participating in regulatory complexes that integrate sugar and organic-acid signaling [27–29]. In petunia, bHLH proteins function as key components of MYB–bHLH–WD40 complexes that coordinate vacuolar acidification with pigment biosynthesis [4, 30–33]. In citrus, the importance of bHLH-mediated regulation is exemplified by Noemi, an AN1-like bHLH transcription factor whose loss-of-function mutations confer the characteristic acidless phenotype [30]. Together, these studies establish bHLH factors as key transcriptional regulators of fruit acidity. Building on this framework, our results identify *CsbHLH113* as a bHLH transcription factor that directly links transcriptional regulation to a defined downstream effector involved in citrate accumulation. CsbHLH113 directly binds the *CsMAC9F1* promoter and activates its transcription (Figs 5 and 6). Genetic manipulation across multiple systems further supports this regulatory relationship: overexpression of *CsbHLH113* increased citrate accumulation in citrus calli and in heterologous tomato systems, whereas VIGS-mediated silencing in kumquat reduced citrate levels (Fig. 7A–I; Fig. S6). Consistently, *CsMAC9F1* transcript abundance closely tracked CsbHLH113 activity, and epistasis analysis indicates that *CsMAC9F1* is required for *CsbHLH113*-driven citrate accumulation (Fig. 7J–L; Fig. S7). Together, these findings support a model in which *CsbHLH113* promotes citrate accumulation predominantly through activation of its target gene *CsMAC9F1*.

Collectively, our findings establish a regulatory model in which the transcription factor CsbHLH113 activates the plasma membrane–localized gene *CsMAC9F1*, thereby adding a previously underappreciated branch to the citrus citrate regulatory network. Although the biochemical activity of CsMAC9F1 remains to be elucidated, our results suggest that *CsMAC9F1* may influence proton and/or anion dynamics at the plasma membrane, which could modulate cytosolic pH homeostasis and create cellular conditions favorable for vacuolar citrate sequestration (Fig. 8). By revealing this plasma membrane-associated regulatory module, our work not only expands the current framework for citrus acidity formation but also identifies the CsbHLH113–*CsMAC9F1* pathway as a distinct route contributing to citrate accumulation and fruit acidity.

**Figure 8:**
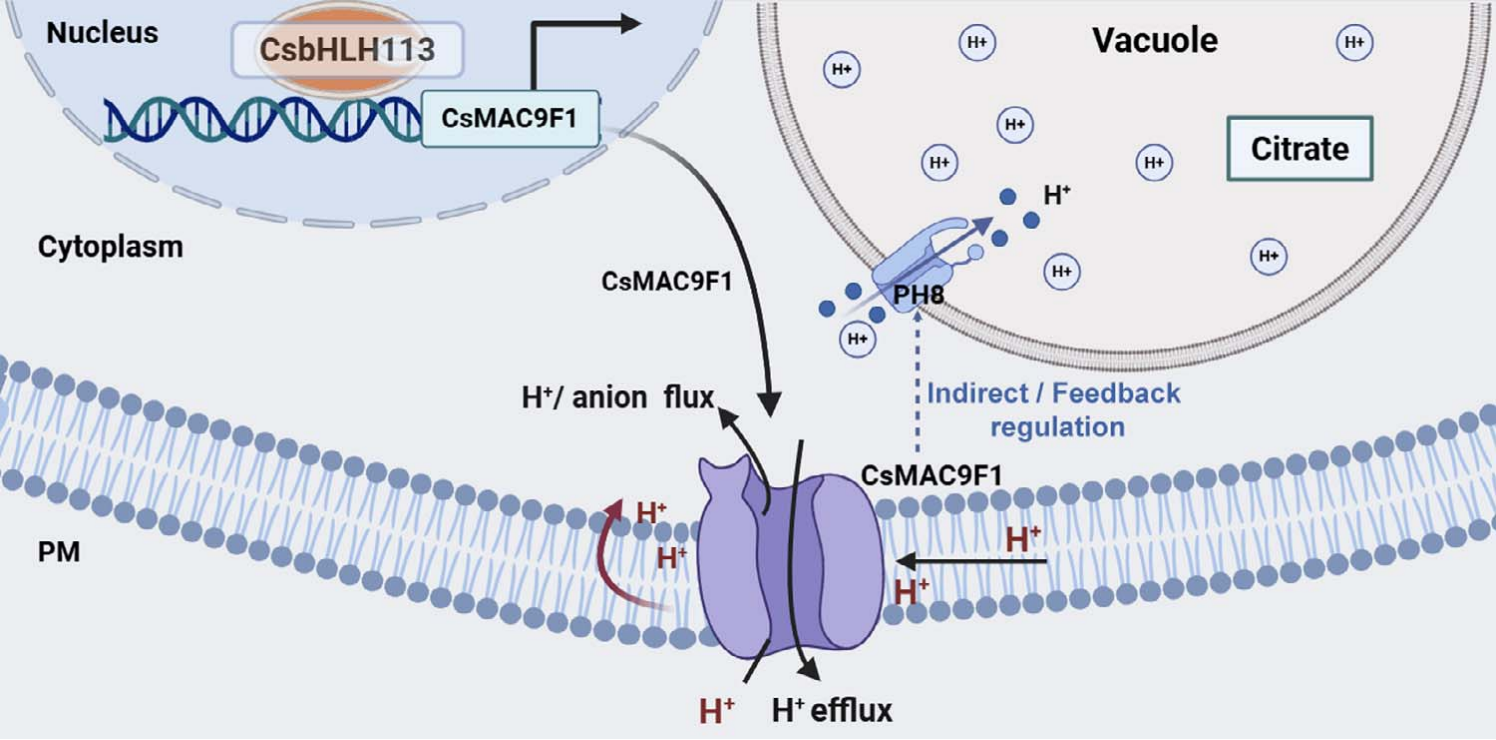
Working model of *CsbHLH113*–*CsMAC9F1*-mediated citrate accumulation in citrus fruit.

## Materials and methods

### Plant materials

Low-acid ‘Qianyang’ Bingtang sweet orange (*C. sinensis* Osbeck; BT) and its acidless bud mutant ‘Jinxiu’ Bingtang sweet orange (ABT) were used in this study. The trees were 5 years old and were cultivated at the Huaihua Comprehensive Experiment Station (Hunan Province, China). Fruit flesh from both cultivars was collected during the fruit expansion stage (90–150 DAFB) in the 2024 growing season for organic acid quantification and gene expression analysis. Samples were immediately frozen in liquid nitrogen and stored at −80°C until use. For each biological replicate, flesh tissues from five fruits were pooled, and at least three independent biological replicates were analyzed for each experimental group.

Citrus calli used for genetic transformation were initiated from ovular tissues of Bingtang sweet orange and maintained on MT solid medium (Murashige and Tucker) supplemented as indicated in the corresponding experiments. Calli were cultured at 25 ± 1°C under a 16-h light/8-h dark photoperiod (40 μmol m^−2^ s^−1^) and subcultured every 20 days.

Kumquat (*Fortunella crassifolia*) trees were grown under standard field conditions at the experimental station of Hunan Agricultural University (Changsha, China). VIGS assays were performed at the green-mature stage of fruit development.

Tomato ‘Micro-Tom’ (*Solanum lycopersicum*) and tobacco (*N. benthamiana*) plants were grown in controlled-environment chambers at 25°C/18°C (day/night), 70% relative humidity, and long-day conditions (16 h light/8 h dark), with a photosynthetic photon flux density of 100 μmol m^−2^ s^−1^ provided by cool-white fluorescent lamps.

### Organic acid and pH value determination

Total soluble solids (TSSs; °Brix) and titratable acidity (TA; %) were determined using a portable fruit quality analyzer (ATAGO PAL-BX/ACID1, Japan). For TA measurement, juice was diluted 50-fold before analysis. Each biological replicate consisted of a composite sample prepared from five randomly selected fruits. For each sample, three independent biological replicates and three technical replicates were performed.

Organic acids were quantified by high-performance liquid chromatography (HPLC) using a Shimadzu LC-20AT system (Japan) equipped with a ultraviolet (UV) detector. The chromatographic conditions were: detection at 210 nm; mobile phase, acetonitrile:phosphate buffer = 0.024:0.576 (v/v); flow rate, 0.6 ml min^−1^; column temperature, 17°C; injection volume, 8 μl. Citrate content was calculated based on fresh weight (FW) and expressed as mg g^−1^ FW.

Fruit pH was measured using a calibrated pH meter (Mettler Toledo FE28, Switzerland). Briefly, 0.3 g of homogenized fruit powder was suspended in 3 ml distilled water prior to measurement. Three biological replicates, each with three technical replicates, were analyzed.

### RNA sequencing and data analysis

Total RNA was extracted from fruit flesh using the SteadyPure Plant RNA Extraction Kit (Accurate Biology, Changsha, China). RNA integrity was assessed using an Agilent 2100 Bioanalyzer, and only samples with an RNA integrity number (RIN) > 7.0 were used for library preparation. Libraries were constructed using the NEBNext Ultra RNA Library Prep Kit (NEB, USA) and sequenced on the BGISEQ-500 platform (LingEn Bio, China).

Raw reads were filtered with fastp v1.0.0 to remove adapters and low-quality reads. Clean reads were aligned to the sweet orange reference genome (*C. sinensis* v1.0; http://citrus.hzau.edu.cn/) using HISAT2 v2.2.1. Gene-level read counting was performed using featureCounts v2.0.1, and differential expression analysis was performed using DESeq2 v1.34.0. Genes with |log₂(fold change)| ≥ 1 and adjusted *P*-value <0.05 (Benjamini–Hochberg correction) were considered differentially expressed.

### Real-time quantitative PCR

Total RNA was treated with DNase I and reverse-transcribed using the Evo M-MLV Reverse Transcription Kit (Accurate Biology, Changsha, China). Real-time quantitative PCR (RT-qPCR) was performed on a Bio-Rad CFX96 system (USA) using SYBR Green Premix. The citrus *ACTIN* gene was used as an internal control. Each assay included three biological replicates, each with three technical replicates. Primer sequences are listed in Table S5.

### Sequence collection, alignment, phylogeny, and structure prediction

Protein sequences of MAC9F1 homologs were retrieved from publicly available databases and genome annotations. In citrus, *CsMAC9F1* was defined as gene Cs1g26060 in the sweet orange reference genome (C. sinensis v1.0; http://citrus.hzau.edu.cn/) and this Gene ID was used for RNA-seq–based expression analyses; the corresponding protein sequence used for alignment and phylogenetic analysis was KAH9710771.1 (*C. sinensis*). Homologous protein sequences from other species were collected using the following accessions/gene model identifiers: AtMAC9F1 (NP_565423.1, *Arabidopsis thaliana*), MdMAC9F1 (XP_008348088.1, *Malus domestica*), OgMAC9F1 (XP_052151793.1, *Oryza glaberrima*), SlMAC9F1 (XP_004232525.1, *S. lycopersicum*), and PhMAC9F1 (Peaxi162Scf00328g00118.1, *Petunia axillaris* gene model). Putative conserved domain boundaries were inferred based on predicted structures available in the AlphaFold Protein Structure Database (https://alphafold.ebi.ac.uk/). Multiple sequence alignment was performed in MEGA12 using ClustalW with default parameters. A Neighbor-Joining phylogenetic tree was constructed in MEGA12 with 1000 bootstrap replicates.

### Overexpression vector construction

The full-length coding sequences (CDSs) of *CsMAC9F1* and *CsbHLH113* were amplified from BT cDNA and cloned into the pBI121-EGFP vector under the control of the CaMV 35S promoter. All constructs were verified by Sanger sequencing and introduced into *Agrobacterium tumefaciens* strain EHA105.

### Plant transformation

*Agrobacterium*-mediated transformation of citrus calli was performed as previously described with modifications [34]. Calli were co-cultivated with *Agrobacterium* (OD_600_ = 0.6) at 20°C in the dark for 3 days and then transferred to selection medium containing 50 mg L^−1^ kanamycin and 400 mg L^−1^ cefotaxime. For kanamycin resistance selection, resistant calli were selected on MT medium containing kanamycin and subcultured for further analysis.

The pBI121-*CsbHLH113* construct was introduced into tomato by *Agrobacterium*-mediated transformation. Transgenic plants were screened by kanamycin resistance (50 mg L^−1^) and PCR genotyping. Homozygous T_1_ plants were grown in chambers at 25°C under a 16-h photoperiod with a light intensity of 120 μmol m^−2^ s^−1^ for citrate quantification and phenotypic analysis.

Transient transformation of citrus juice sacs was performed as described previously with modifications [35]. Mature pomelo (*C. grandis* cv. ‘Longdu Zaoxiang’) fruits were collected on the day of the experiment. Fruits were surface-sterilized with 75% ethanol for 30 s and 1% (w/v) sodium hypochlorite for 15 min, followed by rinsing with sterile water. Juice sacs were isolated, and pedicels were removed. The sacs were immersed in *A. tumefaciens* suspensions carrying the indicated constructs, gently agitated for 5 min, and incubated in the dark for 25 min. After infection, sacs were blotted dry and placed on MS solid medium supplemented with 0.1% agar and 0.1% cefotaxime, overlaid with sterile filter paper. Samples were incubated at 28°C in the dark for 6 days.

### RNAi constructs

A gene-specific fragment (300–400 bp) of *CsMAC9F1* was cloned into the pHGRV vector using Gateway technology (Thermo Fisher Scientific, USA; see Table S5). The resulting construct was introduced into *A. tumefaciens* EHA105. Suspension-cultured calli were filtered and incubated in MT-sucrose liquid medium containing *Agrobacterium* (OD_600_ = 0.6). Hygromycin-resistant calli were selected and validated by RT-qPCR.

### Virus-induced gene silencing in kumquat

For VIGS, gene-specific fragments of *CsMAC9F1* and *CsbHLH113* were cloned into the pTRV2 vector. To ensure silencing specificity, the fragment for CsMAC9F1-VIGS was 154 bp in length, corresponding to nucleotides +1 to +154 relative to the translational start codon (ATG), and the fragment for CsbHLH113-VIGS was 321 bp in length, corresponding to nucleotides +30 to +350 relative to the ATG. *Agrobacterium* cultures harboring pTRV1 and the corresponding pTRV2 constructs were mixed at a 1:1 ratio (OD_600_ = 0.6 each) and infiltrated into kumquat fruit pericarp (0.1–0.3 cm depth; 0.2 ml per fruit) using a needleless syringe following an optimized protocol [36]. Fruits were maintained at 22°C for 15 days before sampling. Silencing efficiency was evaluated by RT-qPCR using three biological replicates (9–15 fruits in total).

### Subcellular localization

The CsMAC9F1–EGFP and CsbHLH113–EGFP fusion constructs were introduced into EHA105 and transiently expressed in *N. benthamiana* leaves by syringe infiltration. At 48 h postinfiltration, fluorescence signals were examined using a confocal laser scanning microscope. EGFP was excited at 488 nm, and chlorophyll autofluorescence was detected with excitation at 633 nm.

### Yeast one-hybrid assay

Two *CsMAC9F1* promoter fragments (−2000 to −1000 bp and −1000 to −1 bp) were cloned into the pAbAi vector. The full-length *CsbHLH113* CDS was cloned into pGADT7. Yeast transformation and interaction assays were performed using the Matchmaker Gold Yeast One-Hybrid System (Clontech) following the manufacturer’s instructions. Transformants were selected on SD/−Ura medium supplemented with 200 ng ml^−1^ Aureobasidin A (AbA).

### Chromatin immunoprecipitation quantitative PCR

Citrus calli overexpressing 35S::*CsbHLH113*-EGFP were used for ChIP-qPCR analysis. The expression of the fusion protein in transgenic callus lines was confirmed by western blotting using an anti-GFP antibody (GeneCreate, Wuhan, China). ChIP assays were performed as previously described [37]. The immunoprecipitated DNA was analyzed by quantitative PCR to determine the enrichment of CsbHLH113 at specific regions of the CsMAC9F1 promoter [38]. All primers used for ChIP-qPCR analysis are listed in Supplementary Table S5.

### Dual LUC reporter assay

The 2-kb CsMAC9F1 promoter was cloned into pGreenII 0800-LUC. The CsbHLH113 CDS was cloned into pBI121-EGFP as the effector. Reporter and effector constructs were co-infiltrated into *N. benthamiana* leaves. Firefly luciferase (LUC) and Renilla luciferase (REN) activities were measured at 48 h postinfiltration using the Dual-Luciferase reporter assay system (Promega) with a spark multifunctional microplate reader (Tecan, Switzerland). Primers used for vector construction are listed in Table S5.

### Figure preparation

The schematic diagram in Fig. 8 was generated using Figdraw (https://www.figdraw.com/).

### Statistical analysis

All data are presented as mean ± standard deviation. Statistical analysis was performed using one-way analysis of variance followed by Tukey’s honestly significant difference (HSD) for pairwise comparisons among groups. Analyses were conducted in SPSS Statistics (version 23.0; SPSS Inc., Chicago, IL, USA).

## Supplementary Material

Web_Material_uhag219

## Data Availability

All datasets associated with this work are presented in the main manuscript and its supplementary data.
