# Supplementary material for: The plasma membrane–localized CsMAC9F1 mediates citrate accumulation in citrus fruit
Source: Hortic Res. 2026 Jun 3;13(11):uhag219. doi: 10.1093/hr/uhag219 (PMC13722863; doi:10.1093/hr/uhag219)
Supplement: Supplementary file 1 [file web_material_uhag219.zip › Supporting Information.docx]

**Supplementary Figure 1| Correlation among ten differentially expressed genes.**

**Supplementary Figure 2| Transient overexpression of *CsMAC9F1* in juice sacs.**

(A) Relative expression levels of *CsMAC9F1* in juice sacs transiently transformed with 35S::CsMAC9F1. Empty vector was used as the control. (B) Citrate content in juice sacs after transient overexpression of *CsMAC9F1*. Data are presented as means ± SD of three biological replicates. Asterisks indicate significant differences compared with the control (**P* < 0.05; ***P* < 0.01; ****P* < 0.001.).

**Supplementary Figure 3| Transient silencing of *CsMAC9F1* in citrus calli.**

(A) Relative expression level of *CsMAC9F1* in citrus calli transiently infected with TRV2::CsMAC9F1-1 and TRV2::CsMAC9F1-2. TRV2 was used as the control. (B) Citrate content in citrus calli after transient silencing of *CsMAC9F1*. Data are presented as means ± SD of three biological replicates. Asterisks indicate significant differences compared with the control (**P* < 0.05; ***P* < 0.01; ****P* < 0.001.).

**Supplementary Figure 4| Expression levels of *CsMAC9F1* in *CsbHLH13* stable overexpression calli.**

**Supplementary Figure 5| Phenotypic analysis of CsbHLH113-overexpressing tomato plants.**

(A) Phenotypic observation of CsbHLH113-OE tomato plants and control plants. (B) Plant height of CsbHLH113-OE and control plants at different days after sowing (C) Crown diameter of CsbHLH113-OE and control plants at different DAS. (D) Stem diameter of CsbHLH113-OE and control plants at different DAS.

**Supplementary Figure 6| Expression levels of *CsMAC9F1* in CsbHLH13-silenced kumquat fruits compared to the control. Data are presented as mean ± SD of three biological replicates.**

**Supplementary Figure 7| Expression levels of *CsPH8* and *CsbHLH113* in response to *CsMAC9F1* manipulation.**

(A, B) Relative expression levels of *CsPH8* (A) and *CsbHLH113* (B) in *CsMAC9F1*-overexpressed citrus seedlings compared to wild-type (WT) controls. (C, D) Relative expression levels of *CsPH8* (C) and *CsbHLH113* (D) in *CsMAC9F1*-silenced citrus calli compared to empty vector (EV) controls.
